# Flow cytometry can reliably capture gut microbial composition in healthy adults as well as dysbiosis dynamics in patients with aggressive B-cell non-Hodgkin lymphoma

**DOI:** 10.1080/19490976.2022.2081475

**Published:** 2022-05-29

**Authors:** Maren Schmiester, René Maier, René Riedel, Pawel Durek, Marco Frentsch, Stefan Kolling, Mir-Farzin Mashreghi, Robert Jenq, Liangliang Zhang, Christine B. Peterson, Lars Bullinger, Hyun-Dong Chang, Il-Kang Na

**Affiliations:** a Department of Hematology, Oncology and Tumor Immunology, Charité-Universitätsmedizin Berlin, Corporate member of Freie Universität Berlin and Humboldt-Universität zu Berlin, Berlin, Germany; b Berlin Institute of Health at Charité, Universitätsmedizin Berlin, Berlin, Germany; c Deutsches Rheuma-Forschungszentrum Berlin (DRFZ), an Institute of the Leibniz Association, Berlin, Germany; d BIH Center for Regenerative Therapies (BCRT), Berlin Institute of Health at Charité – Universitätsmedizin Berlin, Berlin, Germany; e Berlin School of Integrative Oncology, Berlin, Germany; f (DRFZ), an Institute of the Leibniz AssociationTherapeutic Gene Regulation, Deutsches Rheuma-Forschungszentrum, Berlin, Germany; g Department of Genomic Medicine, the University of Texas MD Anderson Cancer Center, Houston, TX, USA; h Department of Population and Quantitative Health Sciences, Case Western Reserve University, Cleveland, OH, USA; i Department of Biostatistics, the University of Texas MD Anderson Cancer Center, Houston, TX, USA; j German Cancer Research Center (DKFZ), Heidelberg, Germany; k German Cancer Consortium (DKTK), Berlin, Germany; l Institute of Biotechnology, Technische Universität Berlin, Germany; m ECRC Experimental and Clinical Research Center, Charité – Universitätsmedizin Berlin, Corporate Member of Freie Universität Berlin and Humboldt Universität zu Berlin, Berlin, Germany

**Keywords:** Flow cytmetry, microbiome, B-cell non-Hodgkin lymphoma, longitudinal, dysbiosis

## Abstract

Modulation of commensal gut microbiota is increasingly recognized as a promising strategy to reduce mortality in patients with malignant diseases, but monitoring for dysbiosis is generally not routine clinical practice due to equipment, expertise and funding required for sequencing analysis. A low-threshold alternative is microbial diversity profiling by single-cell flow cytometry (FCM), which we compared to 16S rRNA sequencing in human fecal samples and employed to characterize longitudinal changes in the microbiome composition of patients with aggressive B-cell non-Hodgkin lymphoma undergoing chemoimmunotherapy. Diversity measures obtained from both methods were correlated and captured identical trends in microbial community structures, finding no difference in patients’ pretreatment alpha or beta diversity compared to healthy controls and a significant and progressive loss of alpha diversity during chemoimmunotherapy. Our results highlight the potential of FCM-based microbiome profiling as a reliable and accessible diagnostic tool that can provide novel insights into cancer therapy-associated dysbiosis dynamics.

## Introduction

In recent years, many studies on the human gut microbiome have linked states of dysbiosis to various changes in human health.^1,2^ For malignant diseases, mounting evidence suggests a complex involvement of the commensal gut microbiota in the formation and progression of neoplasms and in the effectiveness of antineoplastic therapy.^3,4^ The microbial composition has been associated with response to immunotherapy in malignant melanoma and with the incidence of infectious complications during chemotherapy.^5–8^ Furthermore, a loss of microbial diversity correlates with mortality in patients undergoing hematopoietic stem cell transplantation (HSCT).^9,10^

Sequencing approaches are primarily used for characterizing the gut microbiome, allowing for taxonomic identification of organisms and subsequent evaluation of microbial diversity. Even with novel third-generation sequencing approaches reducing costs and turnaround time, molecular microbiome analyses are usually limited to research settings due to specialized equipment and expertise required.^11^ Flow cytometry (FCM), on the other hand, is well established in many laboratories, making it an attractive alternative for microbiome diagnostics.

FCM represents microbes’ morphologies by detecting light scatter from cell shape and subcellular structures. Fluorescent dyes targeting various biomolecules, such as nucleic acids, can be added for further characterization. In contrast to sequencing analysis, the technology allows for multivariate phenotyping, aggregating morphological and physiological characteristics of thousands of single cells into a fingerprint of the studied microbial community.^12^ Cytometric fingerprints can be used to calculate phenotypic diversity metrics akin to their well-established taxonomic counterparts obtained from sequencing analysis, with alpha diversity describing the richness and/or evenness of the examined microbial community and beta diversity assessing the differences in community composition between samples. Cytometric profiles have been used to study changes in microbial composition in various ecological settings and murine models,^13–15^ but the technology has rarely been applied to human samples to date.^16,17^ As computational tools for the analysis of phenotypic microbiome profiles obtained from FCM are becoming more and more refined, even encompassing species recognition, they are paving the way for an exciting and accessible diagnostic tool with great potential for routine clinical application.^18,19^

The gut microbiome of patients with lymphoma disease receiving therapy other than HSCT remains poorly characterized. Aggressive B-cell non-Hodgkin lymphomas (B-NHL) are the most common lymphoid neoplasms in adults. In the last decade, incidence has increased due to demographic shifts. Despite progress in treatment strategies, only about 2/3 of patients can be cured.^20,21^ Thus, there is a clinical need for the identification of factors contributing to the highly aggressive behavior of some B-NHL and of actionable targets for new therapeutic interventions. An involvement of gut microbiota in lymphomagenesis has been described for some lymphoma subtypes, e.g., the strong association of *Helicobacter* infection with mucosal associated lymphoid tissue lymphoma. Furthermore, results from murine models indicate that commensal bacteria can affect lymphomagenesis by altering the systemic inflammatory state.^22,23^ Overall, these findings suggest microbial influence on the course of the disease.^24^

In this study, we set out to evaluate the performance of cytometric microbiome profiling for dysbiosis monitoring in a cohort of healthy controls and B-NHL patients. We validated cytometric findings by 16S rRNA gene sequencing to assess FCM’s capability for rapid and reliable microbial diversity analysis in a clinical setting. This approach allowed for a multimodal, longitudinal characterization of gut microbiome composition and dynamics in patients with B-NHL undergoing non-myeloablative chemoimmunotherapy, supporting patient risk stratification regarding infection susceptibility and disease outcome.

## Results

### Study population

We analyzed a total of 104 fecal samples using flow cytometry and 16S rRNA gene sequencing. 74 of those samples were obtained longitudinally from 12 patients with B-NHL at the time of diagnosis, prior to administration of a therapy cycle, and every three months during a 6-month follow-up period. We obtained between 2 and 11 fecal samples per patient (mean = 5, SD = 3). Blood samples for white blood count analysis were obtained at the same timepoints. All patients received standard first-line treatment with 6 cycles of CHOP backbone (Cyclophosphamide (C), Doxorubicin (H), Vincristine (O) and Prednisone (P)) + anti-CD20 therapy followed by 2 cycles of anti-CD20 therapy alone. No patient had received antibiotic therapy at baseline. Prophylactic antibiotic treatment was administered at physicians’ discretion for the 21 days between therapy cycles. In total, 46 (62%) fecal samples were obtained during treatment with either ciprofloxacin or sulfamethoxazole-trimethoprim alone or in combination. In addition, fecal samples from 30 healthy controls were collected, including age- and sex-matched healthy controls for baseline patient samples. Clinical characteristics of our study population are shown in Table 1.

**Table 1.** Baseline and clinical characteristics of the study population

Patients	n = 12	
Age		
median	56	
range	27–71	
Sex (female)	4 (33.3%)	
Histology		
Diffuse large B-cell lymphoma	10 (83.3%)	
Primary mediastinal large B-cell lymphoma	1 (8.3%)	
High-grade follicular lymphoma	1 (8.3%)	
Stage		
I	3 (25%)	
II	2 (16.7%)	
III	1 (8.3%)	
IV	6 (50%)	
Charlson Comorbidity Index		
median	3	
range	2–7	
Total samples acquired	74	
range	2–11	
Samples collected during prophylactic antibiotic intake	46 (62.2%)	
Ciprofloxacin	3 (4.1%)	
Sulfamethoxazole-trimethoprim	28 (37.8%)	
Ciprofloxacin and sulfamethoxazole-trimethoprim	15 (20.3%)	
Total therapy cycles with episodes of neutropenic fever	3 (4.1%)	
Matched healthy controls	**n = 10**	
Age		
median	56	
range	26–71	
Sex (female)	4 (40%)	
All healthy controls	**n = 30**	
Age		
median	59	
range	26–88	
Sex (female)	22 (73%)	

### Phenotypic microbial diversity is correlated to taxonomic microbial diversity

**Figure 1.**
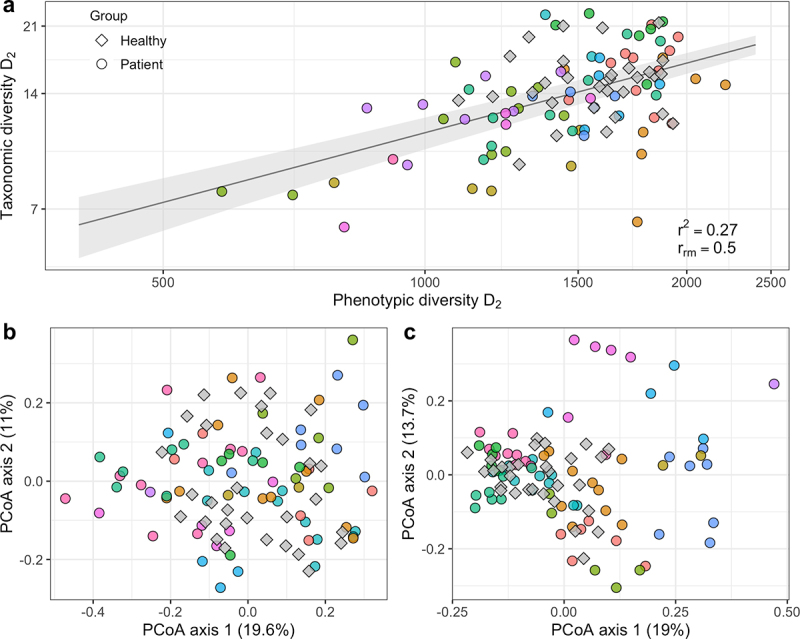
Association between phenotypic and taxonomic diversity. Colors indicate individual patients. (a) Relationship between taxonomic and phenotypic alpha diversity in a B-NHL cohort (inverse Simpson index, D2, depicted on a log scale). The shaded area represents the standard error around the linear mixed effects model. *r^2^*: Nakagawa’s conditional coefficient of determination. *r_rm_*: Repeated measures correlation coefficient. (b) Phenotypic and (c) taxonomic beta diversity (PCoA of Bray-Curtis dissimilarity) analysis of microbial community structures.

Phenotypic diversity analyses were performed using the Phenoflow algorithm.^25^ The inverse Simpson index (D_2_) was determined for alpha diversity analyses, which takes community richness and evenness into account. Taxonomic D_2_ corresponds to the *effective number of species* required in equal abundance to obtain the observed diversity.^26^ Phenotypic D_2_ is calculated in the same manner but must be interpreted in arbitrary units. Using all acquired samples, we compared the phenotypic alpha diversity metrics obtained from flow cytometry analysis to their taxonomic, 16S rRNA gene sequencing-based counterparts. The linear mixed effect model confirmed a statistically significant positive association between taxonomic and phenotypic alpha diversity (*p* = 1.7e-07, repeated measures correlation r*_rm_* = 0.5, *r^2^* = 0.27) (Figure 1a).

As there is no single metric capturing beta diversity, we compared phenotypic and taxonomic beta diversity by relating the microbial community structures detected by the two methods. Procrustes analyses confirmed that both approaches were significantly correlated in terms of ordination results (*p* = .001) (Figure 1(b,c)).

### No alterations of phenotypic microbial diversity in patients with B-NHL at the time of diagnosis

**Figure 2.**
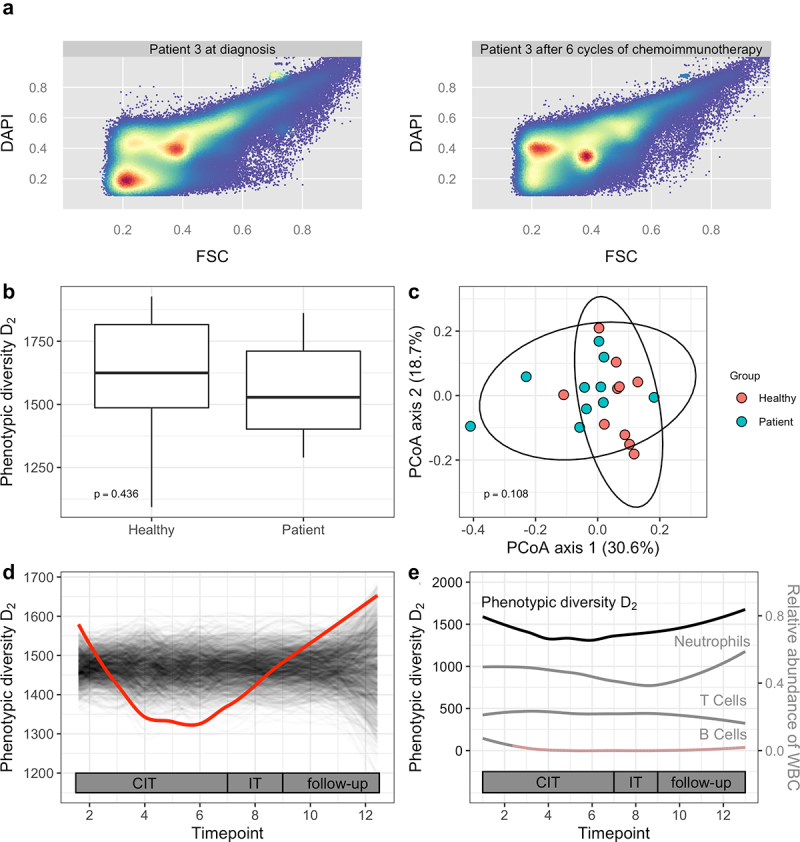
Phenotypic microbial diversity in a B-NHL cohort. (a) Representative flow cytometry plots depicting bacterial DNA content (DAPI, y-axis) and forward scatter (FSC, x-axis) of samples obtained at the time of diagnosis (left) and after the 6^th^ cycle of chemoimmunotherapy (right) from an individual patient. Fluorescence values are arcsinh-transformed and normalized by the maximum fluorescence intensity. The color intensity is proportional to the log-scaled density. (b) Comparison of phenotypic alpha diversity between patients with B-NHL at the time of diagnosis with age- and sex-matched healthy controls. p-value from the corresponding Wilcoxon rank-sum test is shown. (c) PCoA of Bray-Curtis dissimilarity for the comparison of phenotypic beta diversity between patients with B-NHL at the time of diagnosis with age- and sex-matched healthy controls. p-value from the corresponding PERMANOVA is shown. (d) Temporal dynamics of phenotypic alpha diversity in patients with B-NHL during chemoimmunotherapy and follow-up. trendyspliner analysis shows that the group spline for phenotypic alpha diversity (inverse Simpson index, D2) follows a non-linear trend over time that is significantly distinct from the temporally permuted data. The red line represents the true group spline, the gray lines represent the temporally permuted splines under 999 random permutations. (e) Temporal dynamics of phenotypic alpha diversity and white blood cell (WBC) subgroups in patients with B-NHL during chemoimmunotherapy and follow-up. Red shading indicates a depletion below normal WBC range. CIT: chemoimmunotherapy, IT: immunotherapy.

Using the flow cytometry approach, we next set out to characterize the composition and longitudinal shifts of the gut microbiome in our B-NHL cohort. Two representative flow cytometry plots used for cytometric fingerprinting are shown in Figure 2a. We first evaluated for dysbiosis in patients with B-NHL prior to chemoimmunotherapy. No difference in phenotypic alpha diversity between therapy-naïve patients and sex- and age-matched healthy controls was observed, indicating similar richness and evenness components in both groups (Figure 2b, Wilcoxon rank-sum test, *p* > .05). Phenotypic beta diversity, reflecting the variation of community composition between samples, was assessed by principal coordinate analysis (PCoA) of the Bray-Curtis dissimilarity. We found no differences in phenotypic beta diversity between patients and healthy controls (Figure 2c, PERMANOVA, *p* > .05).

### Patients with B-NHL experience a progressive loss of phenotypic alpha diversity during chemoimmunotherapy

Next, we analyzed the dynamics of phenotypic alpha diversity during the course of chemoimmunotherapy and follow-up using a spline-based approach optimized for longitudinal data (Figure 2d).^27^ Phenotypic alpha diversity in patients with B-NHL (red line) exhibited a trend that was statistically significantly distinct from the zero-change distribution formed by temporally permuted data (gray lines) (trendyspliner, *p* = .008). The trajectory of the group spline indicates a loss of microbial richness and evenness during chemoimmunotherapy administration, followed by recovery of diversity during the immunotherapy and follow-up period. To assess for an association between peripheral immune cell and dysbiosis dynamics, contemporaneous analysis of white blood cells was performed. Results indicated normal neutrophil granulocyte and T cell counts throughout the entire study period, which was expected as sampling occurred immediately prior to the administration of therapy cycles at the time of adequate blood count reconstitution. Unlike neutrophil granulocytes, T cells and microbial alpha diversity, B cell depletion remained stable into follow-up due to anti-CD20 therapy (Figure 2e). To evaluate the impact of patients’ clinical characteristics and of prophylactic antibiotic therapy on microbial diversity, we used linear mixed-effects models adjusted for age, sex and sampling timepoint. We observed no associations between phenotypic alpha diversity and comorbidities (Charlson Comorbidity Index) or stage of lymphoma disease. However, lower phenotypic alpha diversity was significantly associated with prophylactic antibiotic treatment (β = −138.8, standard error = 56.3, *p* = .02).

### Confirmation of phenotypic diversity dynamics with 16S rRNA gene sequencing

**Figure 3.**
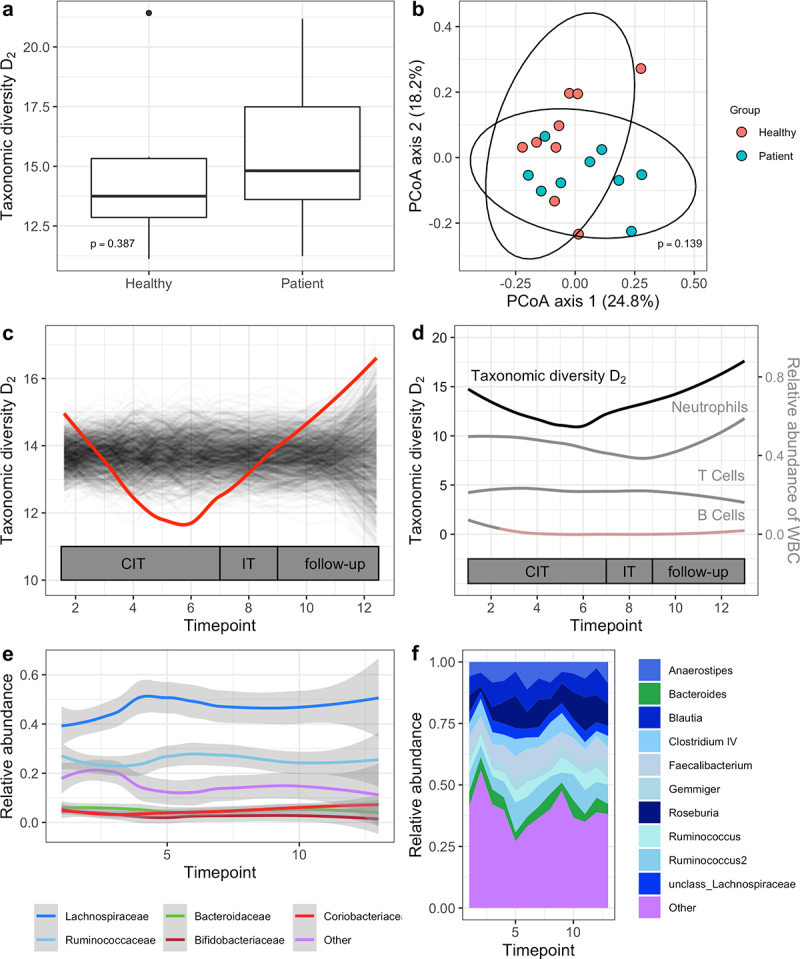
Taxonomic microbial diversity in a B-NHL cohort. (a) Comparison of taxonomic alpha diversity between patients with B-NHL at the time of diagnosis with age- and sex-matched healthy controls. p-value from the corresponding Wilcoxon rank-sum test is shown. (b) PCoA of Bray-Curtis dissimilarity for the comparison of taxonomic beta diversity between patients with B-NHL at the time of diagnosis with age- and sex-matched healthy controls. p-value from the corresponding PERMANOVA is shown. (c) Temporal dynamics of taxonomic alpha diversity in patients with B-NHL during chemoimmunotherapy and follow-up. trendyspliner analysis shows that the group spline for taxonomic alpha diversity (inverse Simpson index, D2) follows a non-linear trend over time that is significantly distinct from the temporally permuted data. The red line represents the true group spline, the gray lines represent the temporally permuted splines under 999 random permutations. (d) Temporal dynamics of taxonomic alpha diversity and white blood cell (WBC) subgroups in patients with B-NHL during chemoimmunotherapy and follow-up. Red shading indicates a depletion below normal WBC range. CIT: chemoimmunotherapy, IT: immunotherapy. (e) Relative abundance of the top 5 overall most abundant microbial families and (f) the top 10 overall most abundant microbial genera during chemoimmunotherapy and follow up. Phyla membership is color-coded in (e) and (f), with *Firmicutes* subtaxa displayed in shades of blue (*Lachnospiraceae* in dark blue, *Ruminococcaceae* in light blue), *Bacteroidetes* subtaxa displayed in green, *Actinobacteria* subtaxa displayed in shades of red and others displayed in purple.

Using 16S rRNA gene sequencing, we then repeated the microbial diversity analyses for validation of FCM. The taxonomic approach yielded results concurrent with cytometric profiling, with no differences in taxonomic alpha or beta diversity observed between patients at the time of diagnosis and healthy controls (Figure 3(a,b), Wilcoxon rank-sum test for alpha diversity and PERMANOVA for beta diversity, *p* > .05). A significant and progressive loss of taxonomic alpha diversity during the chemoimmunotherapy treatment phase followed by complete reconstitution was noted (trendyspliner, *p* = .04, Figure 3c), discordant to B cell dynamics (Figure 3d). Linear mixed-effects modeling revealed a statistically significant association between lower taxonomic alpha diversity and prophylactic antibiotic intake (β = −2, standard error = 0.7, *p* = .009). No significant associations were observed for comorbidities or stage of lymphoma disease.

### Bacterial taxa dynamics during chemoimmunotherapy

Finally, we examined alterations of the microbial composition in patients with B-NHL during chemoimmunotherapy and follow-up at different taxonomic levels. Specifically, we evaluated the temporal dynamics of the top 3 overall most abundant phyla, the top 5 overall most abundant families and top 10 overall most abundant genera in our cohort for significant non-linear longitudinal trends (Figure 3e,f, Supplementary Tables S1–S3). At the phylum level, *Firmicutes* showed a trend toward an increase in relative abundance during the study period (trendyspliner, *p* = .053). At the family level, an expansion of *Lachnospiraceae* (phylum *Firmicutes*) was noted after the initiation of chemoimmunotherapy (trendyspliner, *p* = .01) with an average abundance of 43% at baseline, a peak at 52% before the administration of the fourth chemoimmunotherapy cycle and a persistent elevation during the rest of the study period, including follow-up (Supplementary Figure S1A). At the genus level, relative abundance of *Roseburia* (family *Lachnospiraceae*, phylum *Firmicutes*) exhibited an increase during the study period (trendyspliner, *p*= .008, Supplementary Figure S1B).

## Discussion

Our current knowledge on the gut microbiome of cancer patients during chemoimmunotherapy is derived from studies using sequencing-based approaches, generally detecting a loss of microbial diversity during antineoplastic treatment and linking dysbiosis to increased mortality.^9,10,28,29^ Diversity analysis by sequencing requires specialized equipment, expertise and pooling of many samples to be cost-effective, impeding its use in routine diagnostics. FCM, on the other hand, constitutes a tool well-suited for everyday clinical use, as it permits rapid and inexpensive testing that can be performed with equipment readily available in most hospital laboratories. We believe that patients could benefit from routine cytometric dysbiosis monitoring to support risk stratification regarding dysbiosis-related complications and allowing for early and targeted countermeasures. With various therapies aiming to reconstitute disrupted microbiomes in cancer patients on their way into clinical practice and first reports suggesting significant benefits,^30–32^ FCM could prove beneficial for their therapeutic stewardship.

We tested cytometric fingerprinting in fecal samples from healthy volunteers and patients with B-NHL, finding robust correlation with both alpha and beta diversity measures obtained from 16S rRNA gene sequencing. This correlation is especially noteworthy considering the distinct data types generated by both methods, each harboring their own potential methodical and computational biases. Even so, FCM consistently yielded results in accordance with16S rRNA gene sequencing for all analyses performed in this study. The longitudinal analysis of patient samples obtained during regular clinical care emphasizes FCM’s capability for reliable monitoring of individual shifts in microbial composition and serves as a proof-of-principle regarding the method’s suitability for integration into routine diagnostics.

Microbial community analysis can also be achieved using whole-genome shotgun sequencing (WGS), which typically provides a higher taxonomic resolution and allows for additional metabolic functional profiling. Further validation comparing FCM’s performance to metagenome sequencing regarding microbial diversity analysis is warranted, but will likely also result in a positive association, as both WGS and 16S rRNA sequencing capture similar trends in diversity metrics.^33^ Indeed, there is already work showing that FCM mirrors alterations in the microbiota profile observed by meta-proteomic approaches for simple model communities and for complex porcine microbiota.^34,35^

Dysbiosis patterns in lymphoma patients have mainly been described in the setting of HSCT,^28,36^ with few reports regarding patients receiving less aggressive therapeutic regimens and a lack of high-resolution longitudinal data. So far, only two studies have investigated gut microbial diversity in patients at the time of lymphoma disease diagnosis. Diefenbach et al. found significant differences in beta diversity between a histologically heterogenous (aggressive and indolent B-cell, T-cell, Hodgkin and unclassified lymphoma) cohort of n = 51 lymphoma patients and healthy controls, but did not show alpha diversity.^37^ In a comparison of n = 25 patients with diffuse large B-cell lymphoma at the time of diagnosis and healthy controls, Yuan et al. did not find differences in alpha diversity, but noted altered microbial compositions in patients regarding beta diversity.^38^ In accordance with this data, we did not observe differences in alpha diversity between treatment-naïve patients and age- and sex-matched healthy controls. However, we also did not find differences in beta diversity. The analysis of larger cohorts is needed to assess the value of microbiota-targeted biomarkers in lymphoma diagnostics prior to therapy initiation. Importantly, both 16S rRNA sequencing and FCM provide the same results, supporting our key finding that flow cytometric microbial diversity analysis is highly feasible.

Associations between the gut microbiome and immune reconstitution have been reported in patients undergoing HSCT, with commensals likely providing nutritional support for hematopoiesis and perhaps affecting the extra- and intravasation of immune cells.^39^ HSCT indues a prolonged and continuous immunosuppression. In contrast, patients in our cohort experienced near complete peripheral immune cell recovery between therapy cycles, as shown for T cells and neutrophil granulocytes, but remained B cell-depleted due to anti-CD20 treatment. In this setting, we noted a progressive decrease in alpha diversity during chemoimmunotherapy, with microbial reconstitution commencing during subsequent immunotherapy and fully reached during follow-up, seemingly unrelated to continued B cell depletion. These findings suggest that B cell and microbial reconstitution are not linked.

During HSCT, sustained loss of microbial diversity driven by intensive chemotherapy and therapy-related factors, such as antimicrobial prophylaxis and nutritional alterations, has been associated with an increased incidence of infectious complications and increased mortality.^7,9,10,40^ The progressive loss of alpha diversity in our cohort suggests that patients in a similar therapeutic setting receiving non-myeloablative therapy are also at risk for dysbiosis-associated complications and could benefit from interventions aiming at microbial modulation. As dysbiosis was aggravated by prophylactic administration of antibiotics, we stress the necessity for careful evaluation of the need for antimicrobial prophylaxis in patients at intermediate risk for neutropenic complications, such as those affected by B-NHL. We note that future studies are needed to delineate the individual contribution of chemoimmunotherapy and antibiotic treatment to dysbiosis.

In line with a recent study in children with acute lymphoblastic leukemia undergoing chemotherapy,^41^ we found a sustained increase in *Lachnospiraceae* in our cohort, with indications of a continuous taxonomic relationship at the phylum and genus level encompassing *Firmicutes* and *Roseburia*. The impact of *Lachnospiraceae* on their hosts’ health remains controversial, and increased abundances have been linked to metabolic, liver and kidney disease. However, some subtaxa (e.g., *Roseburia*) are known to protect the intestinal epithelial barrier by producing short-chain fatty acids and to constrain the production of pro-inflammatory cytokines.^42^ Increased *Lachnospiraceae* abundance has also been associated with a decrease in mortality from graft-versus-host disease in HSCT.^43^ While our sample size is too small to detect this type of association, we do note a low incidence of infectious complications in our cohort. As all of our analyses of taxonomic dynamics are exploratory, further validation is needed.

In conclusion, our study highlights the profound potential of cytometric microbiome profiling of human samples. This high-throughput method rapidly and reliably captured dysbiosis dynamics in a “real-world” setting, thus constituting a promising new tool for routine diagnostics. Furthermore, our results expand upon the current knowledge on longitudinal dysbiosis in B-NHL patients treated with conventional first line chemoimmunotherapy. Additional studies on microbiota dynamics and dysbiosis-related complications in cancer patients are warranted to delineate clinically relevant biomarkers that could guide preventive therapeutic measures.

## Patients and methods

### Study design and sample collection

Fecal and blood samples were collected from n = 12 patients with aggressive B-cell non-Hodgkin lymphoma at the time of diagnosis, before every cycle of chemoimmunotherapy and every three months during follow-up for a period of six months. All patients provided written informed consent for the participation in the study and ethical approval was obtained from the Charité Institutional Review Board (EA4/230/19). In addition, fecal samples were obtained from n = 30 healthy volunteers, including sex- and age-matched healthy controls for each patient with a baseline sample available (*n* = 10). Inclusion criteria for healthy controls were no history of malignant disease or chronic gastrointestinal inflammatory disease and no intake of antibiotics or steroids within the last four weeks. Fecal samples were stored at −80°C until further processing after a maximum storage of 24 h at room temperature for transportation.

### Isolation of fecal bacteria and flow cytometry

The bacterial suspension was fixed with 2% formaldehyde and stored in 70% ethanol at −20°C until further analysis. Staining with 4′,6-diamidino-2-phenylindole (DAPI) was performed as previously described.^15^ Fluorescence beads Fluoresbrite BB Carboxylate microspheres 0.5 μm and 1 µm (Polysciences) were added to each sample as an internal benchmark for standardized measurements, with stability of the bead signals indicating the stability of the instrument and comparability of the samples. Cytometric measurements were performed on a BD Influx Cell Sorter (BD Biosciences). Excitation at 488 nm was used to measure forward scatter (FSC) and a UV-laser (355 nm) to measure DAPI-DNA fluorescence. Calibration of optics and laser performance with eight peak Rainbow Calibration Particles (Spherotech Inc.) was performed at regular intervals during a measurement day. For all measurements, at least 300,000 stained cells were recorded.

### DNA extraction and 16S rRNA gene sequencing

Fecal DNA was isolated using the Quick-DNA Fecal/Soil Microbe Kit (Zymo Research) according to manufactures instructions. 16S rDNA library preparation was performed according to Illumina’s 16S Metagenomic Sequencing Library Preparation protocol using the 16S V3 forward (341 F) and V4 reverse (805 R) primer pair with Illumina adapter overhang nucleotide sequences added. Sequencing was performed using the Miseq Reagent Kit V3 on the Illumina Miseq 2500 platform. Raw data were processed and de-multiplexed using MiSeq Reporter Software. Forward and reverse reads were combined using PANDAseq 2.11 with a minimum overlap of 25 bases and classified using “classifier.jar” 2.13 from the Ribosomal Database Project with a confidence cutoff of 50%.^44–46^ Copy number-adjusted bacterial counts were used for further analysis.

### Phenotypic diversity analysis

Flow cytometry analysis was performed using the Phenoflow R package. Briefly, raw data was exported in .fcs format and denoised using the same strategy for all samples. The phenotypic intensity values were normalized based on the maximum signal intensity of the DAPI parameter. The Diversity_rf() function was used to calculate the inverse Simpson index (D_2_) for phenotypic alpha diversity analysis on the DAPI and FSC parameters using default settings except for an increased grid size of 256 × 256 for kernel density estimation. Phenotypic beta diversity was evaluated by PCoA of Bray-Curtis dissimilarity using the beta_div_fcm() function with default settings.

### Taxonomic diversity analysis

Prior to taxonomic diversity analysis, rescaling to the minimum library size was performed. For taxonomic alpha diversity analysis, D_2_ was calculated for all samples with > 10,000 reads (*n* = 103) with the Diversity_16S() function from the Phenoflow package using default settings. Taxonomic beta diversity was evaluated by PCoA of Bray-Curtis dissimilarity, which was calculated on taxon proportions to allow a direct comparison with phenotypic beta diversity.

### Flow cytometric analysis of whole-blood counts

100 µl of fresh heparinized whole blood were collected on the above-described time points. A mix of 18 fluorochrome-conjugated antibodies (Biolegend, Supplementary Table S4) was added to the whole blood samples and staining was performed in darkness at room temperature. After incubation with erythrocyte lysis buffer (Qiagen), PBS + 0,4% BSA was added and samples were immediately measured using a Cytoflex LX cytometer (Beckman Coulter). A defined volume of 500 µl per sample was measured at a collection rate of 50 µl per second, allowing for direct evaluation of cells per µl whole blood. Flow cytometry data was analyzed using CytExpert Software Version 2.4 (Beckman Coulter). Our gating strategy is shown in Supplementary Figure S2.

### Statistical analyses

Statistical analyses were performed in the R environment (version 4.0.5) using the packages Phenoflow (version 1.1.2), flowViz (version 1.54.0), FlowAI (version 1.20.1), phyloseq (version 1.34.0), splinectomeR (version 0.9.4b), microbiome (version 1.12.0), lmerTest (version 3.1–3), performance (version 0.8.0), rmcor (version 0.4.5), plyr (version 1.8.6), dplyr (version 1.0.5), stats (version 4.0.5), vegan (version 2.5–7), ggplot2 (version 3.3.3), grid (version 4.0.5) and lemon (0.4.5). A linear mixed effect model was calculated to relate phenotypic and taxonomic alpha diversity (log transformed D_2_) including a random effect for study identifier. Differences in alpha diversity between groups were calculated using the Wilcoxon rank-sum test. Differences in beta diversity between groups were calculated with permutational multivariate analysis of variance (PERMANOVA) of the Bray-Curtis dissimilarity using the adonis() function (999 permutations). Procrustes analysis was performed to evaluate for similarities between taxonomic and phenotypic beta diversity analysis (protest() function, 999 permutations). Non-zero changes over time in alpha diversity were evaluated using the trendyspliner() function (999 permutations). The same function was used to analyze non-zero changes over time in relative microbial abundances at the phylum, family, and genus level. Correction for multiple testing was not performed due to the exploratory nature of the analyses. For longitudinal trend analyses, only patients with at least 3 samples were included (*n =* 11). Associations between alpha diversity and clinical characteristics were studied using a linear mixed model approach adjusted for age, sex, and sampling timepoint and including a random effect for patient identifier.

## Supplementary Material

Supplemental Material

## Data Availability

Raw sequence data were deposited at the NCBI Sequence Read Archive (https://www.ncbi.nlm.nih.gov/) under the BioProject accession number PRJNA782417. The flow cytometry data files are deposited in the FlowRepository database (https://flowrepository.org/) with the repository ID FR-FCM-Z4RH.
