# Supplementary material for: Flow cytometry can reliably capture gut microbial composition in healthy adults as well as dysbiosis dynamics in patients with aggressive B-cell non-Hodgkin lymphoma
Source: Gut Microbes. 2022 May 29;14(1):2081475. doi: 10.1080/19490976.2022.2081475 (PMC9154785; doi:10.1080/19490976.2022.2081475)
Supplement: Supplemental Material [file KGMI_A_2081475_SM0295.zip › Supplementary Figure 2.pdf]

A

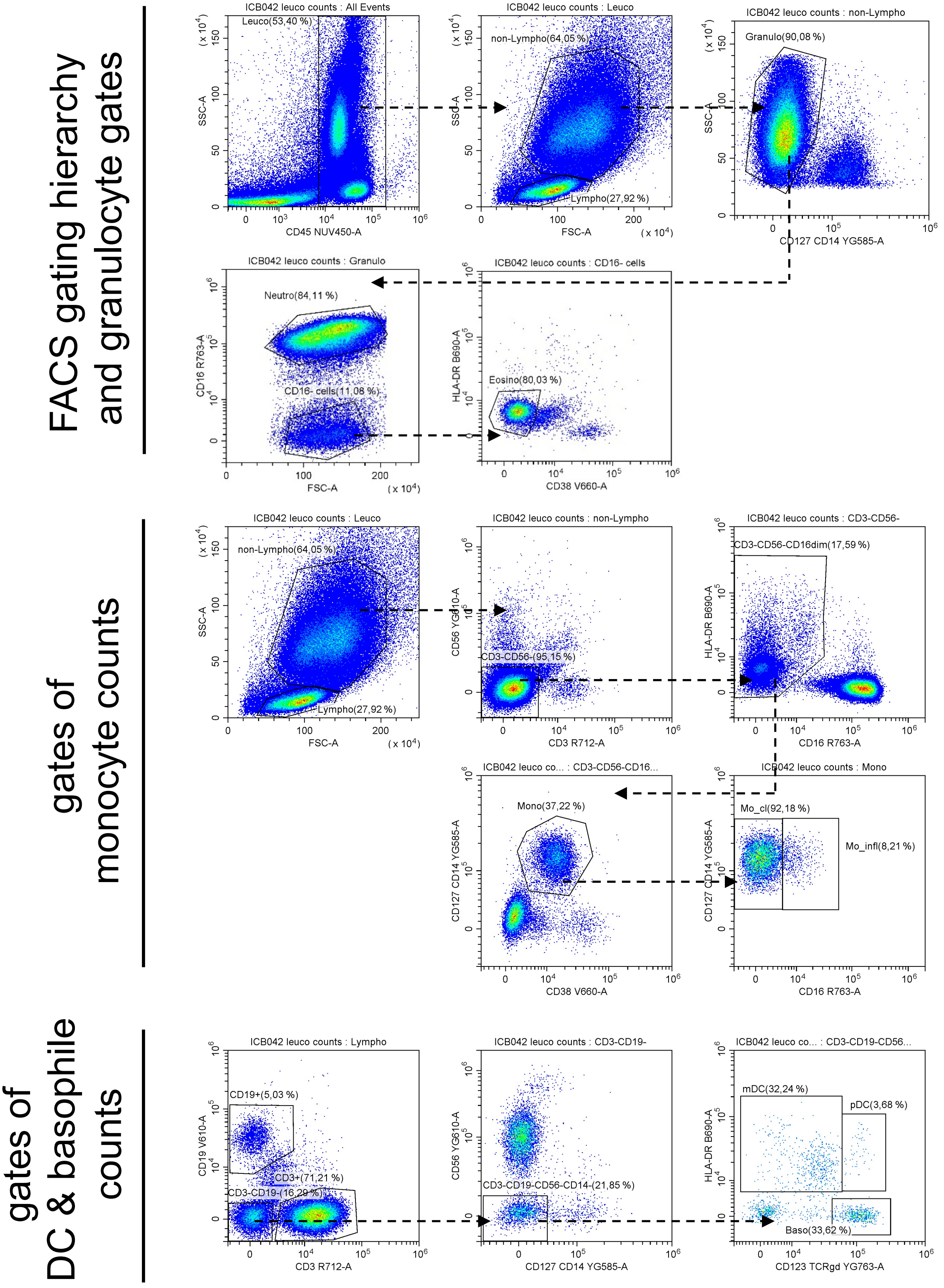

FACS gating hierarchy and granulocyte gates
gates of monocyte counts
gates of DC & basophile counts
ICB042 leuco counts : All Events
Leuco(53,40 %)
CD45 NUV450-A
SSC-A
ICB042 leuco counts : Leuco
non-Lympho(64,05 %)
Lympho(27,92 %)
FSC-A
ICB042 leuco counts : non-Lympho
Granulo(90,08 %)
CD127 CD14 YG585-A
ICB042 leuco counts : Granulo
Neutro(84,11 %)
CD16- cells(11,08 %)
CD16 R763-A
ICB042 leuco counts : CD16- cells
Eosino(80,03 %)
HLA-DR B690-A
CD38 V660-A
ICB042 leuco counts : non-Lympho
CD3-CD56-(95,15 %)
CD56 YG610-A
CD3 R712-A
ICB042 leuco counts : CD3-CD56-
CD3-CD56-CD16dim(17,59 %)
ICB042 leuco co... : CD3-CD56-CD16...
Mono(37,22 %)
ICB042 leuco counts : Mono
Mo_cl(92,18 %)
Mo_infl(8,21 %)
ICB042 leuco counts : Lympho
CD19+(5,03 %)
CD3+(71,21 %)
CD3-CD19-(16,29 %)
CD19 V610-A
ICB042 leuco counts : CD3-CD19-
CD3-CD19-CD56-CD14-(21,85 %)
ICB042 leuco co... : CD3-CD19-CD56...
mDC(32,24 %)
pDC(3,68 %)
Baso(33,62 %)
CD123 TCRgd YG763-A

B

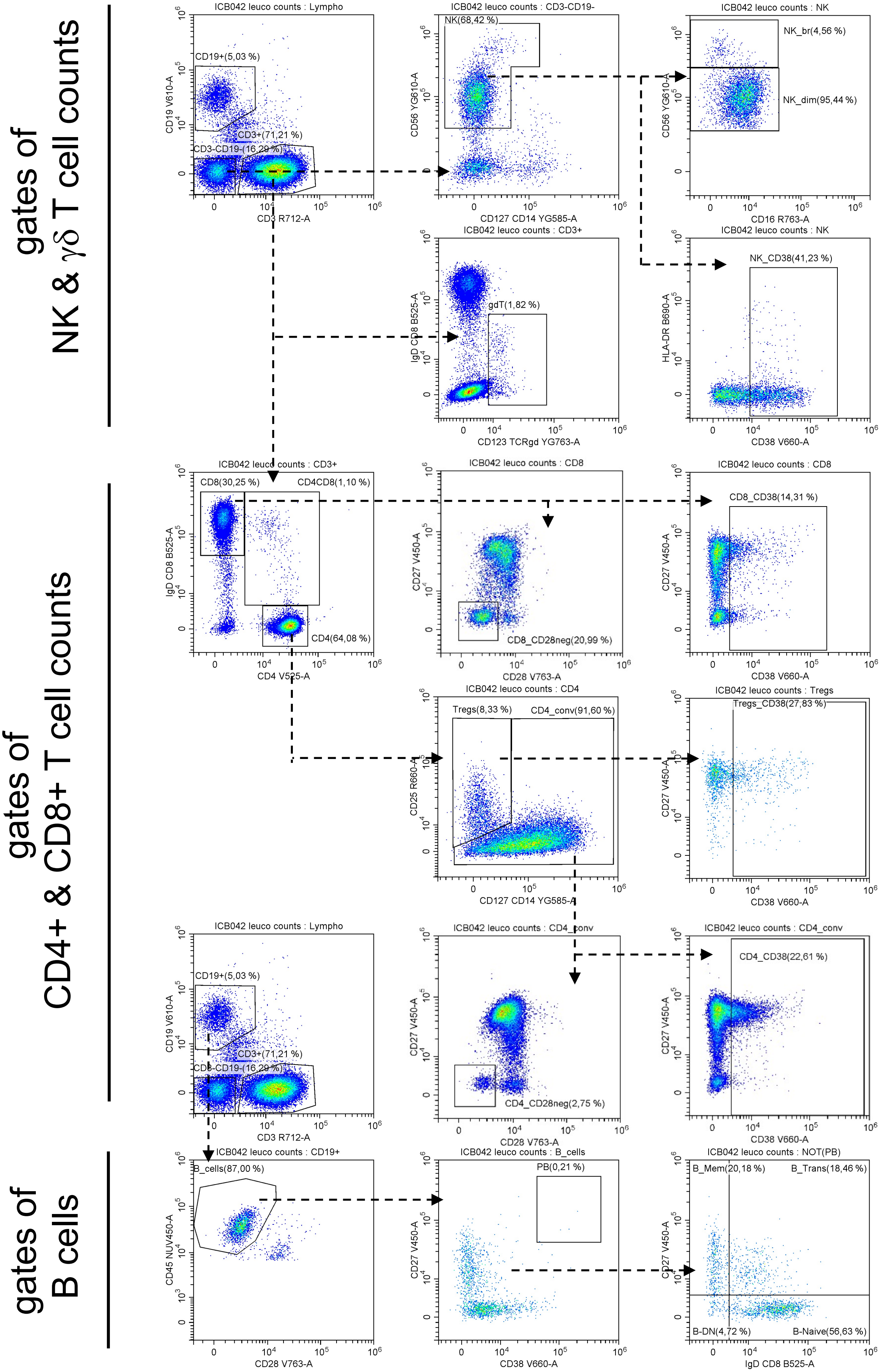

gates of NK & γδ T cell counts
gates of CD4+ & CD8+ T cell counts
gates of B cells
ICB042 leuco counts : Lympho
CD19+(5,03 %)
CD3+(71,21 %)
CD3-CD19-(16,29 %)
CD19 V610-A
CD3 R712-A
ICB042 leuco counts : CD3-CD19-
NK(68,42 %)
CD56 YG610-A
CD127 CD14 YG585-A
ICB042 leuco counts : NK
NK_br(4,56 %)
NK_dim(95,44 %)
CD16 R763-A
ICB042 leuco counts : CD3+
gdT(1,82 %)
IgD CD8 B525-A
CD123 TCRgd YG763-A
NK_CD38(41,23 %)
HLA-DR B690-A
CD38 V660-A
CD8(30,25 %)
CD4CD8(1,10 %)
CD4(64,08 %)
CD4 V525-A
ICB042 leuco counts : CD8
CD8_CD28neg(20,99 %)
CD27 V450-A
CD28 V763-A
CD8_CD38(14,31 %)
ICB042 leuco counts : CD4
Tregs(8,33 %)
CD4_conv(91,60 %)
CD25 R660-A
ICB042 leuco counts : Tregs
Tregs_CD38(27,83 %)
ICB042 leuco counts : CD4_conv
CD4_CD28neg(2,75 %)
CD4_CD38(22,61 %)
ICB042 leuco counts : CD19+
B_cells(87,00 %)
CD45 NUV450-A
ICB042 leuco counts : B_cells
PB(0,21 %)
ICB042 leuco counts : NOT(PB)
B_Mem(20,18 %)
B_Trans(18,46 %)
B-DN(4,72 %)
B-Naive(56,63 %)
