# Supplementary material for: Flow cytometry can reliably capture gut microbial composition in healthy adults as well as dysbiosis dynamics in patients with aggressive B-cell non-Hodgkin lymphoma
Source: Gut Microbes. 2022 May 29;14(1):2081475. doi: 10.1080/19490976.2022.2081475 (PMC9154785; doi:10.1080/19490976.2022.2081475)
Supplement: Supplemental Material [file KGMI_A_2081475_SM0295.zip › Supplementary Tables.docx]

**Supplementary Table 1.** Evaluation for significant non-linear longitudinal trends in top 3 overall most abundant phyla over time using the trendyspliner() function. Raw p-values are reported.

| phylum | p-value |
| --- | --- |
|  |  |
| Actinobacteria | 0.084 |
| Bacteroidetes | 0.542 |
| Firmicutes | 0.053 |

**Supplementary Table 2.** Evaluation for significant non-linear longitudinal trends in top 5 overall most abundant families over time using the trendyspliner() function. Raw p-values are reported.

| family | p-value |
| --- | --- |
|  |  |
| Bacteriodaceae | 0.902 |
| Bifidobacteriaceae | 0.429 |
| Coriobacteriaceae | 0.097 |
| Lachnospiraceae | 0.01 |
| Ruminococcaceae | 0.066 |

**Supplementary Table 3.** Evaluation for significant non-linear longitudinal trends in top 10 overall most abundant genera over time using the trendyspliner() function. Raw p-values are reported.

| genus | p-value |
| --- | --- |
|  |  |
| Anaerostipes | 0.198 |
| Bacteroides | 0.9 |
| Blautia | 0.493 |
| Clostridium IV | 0.051 |
| Faecalibacterium | 0.905 |
| Gemmiger | 0.995 |
| Roseburia | 0.008 |
| Ruminococcus | 0.718 |
| Ruminococcus2 | 0.778 |
| unclass_Lachnospiraceae | 0.168 |

**Supplementary Table 4.** Antibodies used for flow cytometric analysis of whole-blood counts.

| Antibody | Fluorochrome | Clone | Company |
| --- | --- | --- | --- |
| CD25 | APC | [M-A251](https://www.biolegend.com/de-de/search-results?Clone=M-A251) | Biolegend |
| CD16 | APC/F750 | [3G8](https://www.biolegend.com/de-de/search-results?Clone=3G8) | Biolegend |
| CD4 | BV510 | [RPA-T4](https://www.biolegend.com/de-de/search-results?Clone=RPA-T4) | Biolegend |
| CD127 | PE | [A019D5](https://www.biolegend.com/de-de/search-results?Clone=A019D5) | Biolegend |
| TCRγδ | PE/Cy7 | [B1](https://www.biolegend.com/de-de/search-results?Clone=B1) | Biolegend |
| HLA-DR | PerCP | [L243](https://www.biolegend.com/de-de/search-results?Clone=L243) | Biolegend |
| CD28 | BV785 | [CD28.2](https://www.biolegend.com/de-de/search-results?Clone=CD28.2) | Biolegend |
| CD45 | BUV395 | HI30 | BD |
| CD3 | A700 | [UCHT1](https://www.biolegend.com/de-de/search-results?Clone=UCHT1) | Biolegend |
| CD14 | PE | [HCD14](https://www.biolegend.com/de-de/search-results?Clone=HCD14) | Biolegend |
| CD56 | PE/Dazzle | [HCD56](https://www.biolegend.com/de-de/search-results?Clone=HCD56) | Biolegend |
| CD123 | Pe/Cy7 | [6H6](https://www.biolegend.com/de-de/search-results?Clone=6H6) | Biolegend |
| IgD | FITC | [IA6-2](https://www.biolegend.com/de-de/search-results?Clone=IA6-2) | Biolegend |
| CD8 | A488 | [SK1](https://www.biolegend.com/de-de/search-results?Clone=SK1) | Biolegend |
| CD27 | BV421 | [O323](https://www.biolegend.com/de-de/search-results?Clone=O323) | Biolegend |
| CD19 | BV605 | [HIB19](https://www.biolegend.com/de-de/search-results?Clone=HIB19) | Biolegend |
| CD38 | BV650 | [HB-7](https://www.biolegend.com/de-de/search-results?Clone=HB-7) | Biolegend |
| CD33* | BV785 | [WM53](https://www.biolegend.com/de-de/search-results?Clone=WM53) | Biolegend |
