# Supplementary figures and images for: Flow cytometry can reliably capture gut microbial composition in healthy adults as well as dysbiosis dynamics in patients with aggressive B-cell non-Hodgkin lymphoma
Source: Gut Microbes. 2022 May 29;14(1):2081475. doi: 10.1080/19490976.2022.2081475 (PMC9154785; doi:10.1080/19490976.2022.2081475)

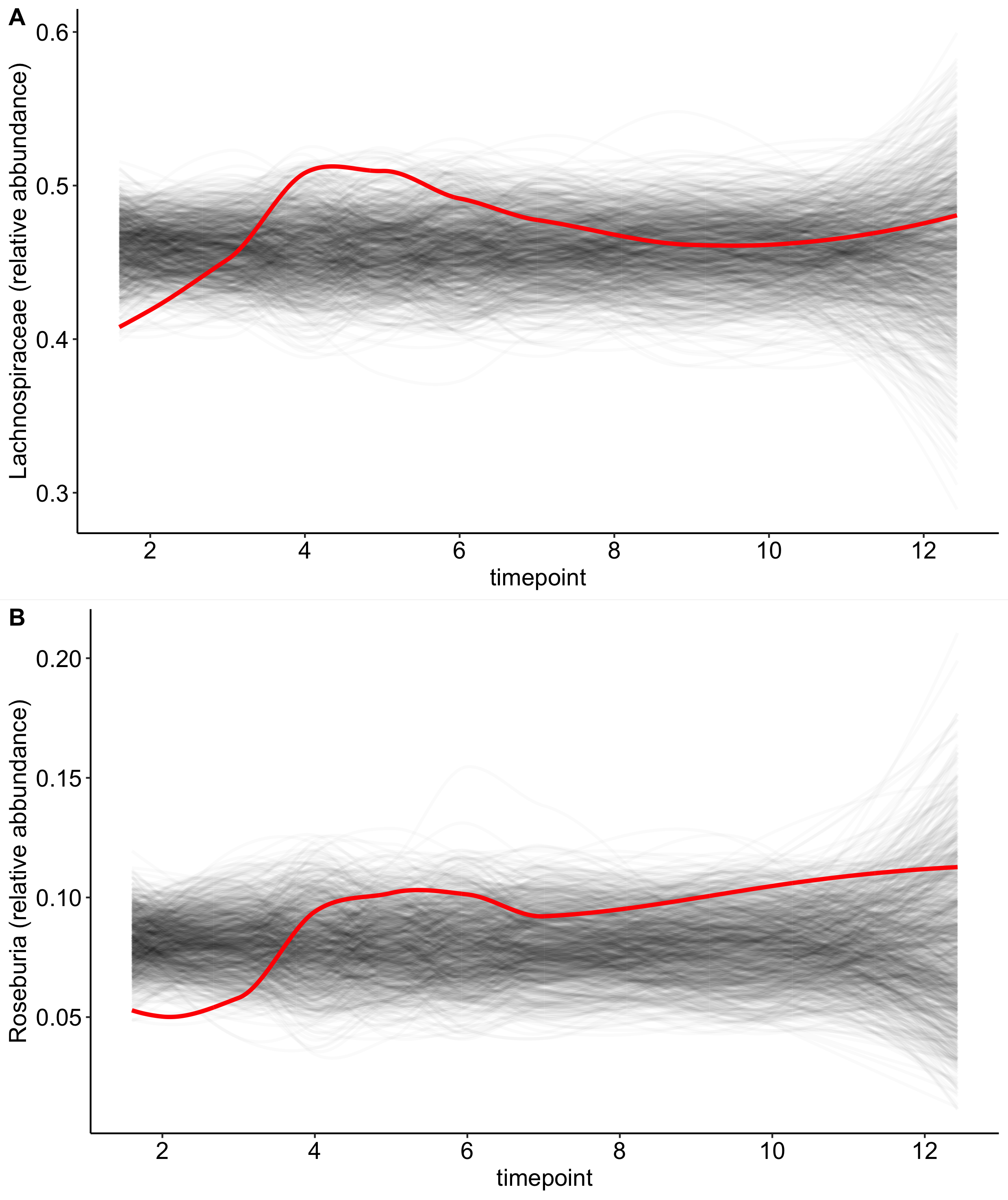

Supplement: Supplemental Material [file KGMI_A_2081475_SM0295.zip › Supplementary Figure 1 revised.tiff]
